# Prognostic benefit of glucagon-like peptide-1 receptor agonists addition to sodium-glucose cotransporter 2 inhibitors in patients with atherosclerotic cardiovascular disease and heart failure: a cohort study

**DOI:** 10.1093/ehjcvp/pvaf014

**Published:** 2025-02-17

**Authors:** Sih-Yao Chen, Jheng-Yan Wu, Kuang-Ming Liao, Yu-Min Lin

**Affiliations:** Division of Cardiology, Department of Internal Medicine, Chi Mei Medical Center, Liouying, Tainan, 736, Taiwan; Department of Nutrition, Chi Mei Medical Center, Tainan 710, Taiwan; Department of Public Health, College of Medicine, National Cheng Kung University, Tainan 704, Taiwan; Department of Internal Medicine, Chi Mei Medical Center, Chiali, Tainan, 722, Taiwan; Min-Hwei Junior College of Health Care Management, Tainan 736, Taiwan; Division of Cardiology, Department of Internal Medicine, Chi Mei Medical Center, Chiali, No.606, Jialixing Jiali Dist., Tainan, 722, Taiwan

**Keywords:** GLP-1RA, SGLT2i, ASCVD, Heart failure, MACE

## Abstract

**Aims:**

Managing patients with atherosclerotic cardiovascular disease (ASCVD) and heart failure (HF) is challenging. While sodium-glucose cotransporter 2 inhibitors (SGLT2i) and glucagon-like peptide-1 receptor agonists (GLP-1 RA) show cardiovascular benefits, the impact of combining these agents is unclear. This study evaluated whether adding GLP-1 RA to SGLT2i provides additional benefits in patients with both ASCVD and HF.

**Methods and results:**

This retrospective observational study utilized the TriNetX database to analyse patients with ASCVD and HF who initiated GLP-1 RA with SGLT2i or SGLT2i alone from 1 August 2016 to 30 September 2024. A total of 2 797 317 patients were identified, with 96 051 patients meeting inclusion criteria. After propensity score matching, 5272 patients in each group were analysed. Primary outcomes included mortality or hospitalization within 1 year; secondary outcomes examined mortality, hospitalization, and heart failure exacerbation (HFE). Patients receiving GLP-1RA and SGLT2i therapies had significantly lower risk of mortality or hospitalization [hazard ratio (HR) 0.78; 95% confidence interval (CI) 0.74–0.83], mortality (HR 0.72; 95% CI 0.62–0.84), hospitalization (HR 0.78; 95% CI 0.73–0.83), and HFE (HR 0.77; 95% CI 0.72–0.83) vs. SGLT2i alone. Subgroup analyses showed consistent benefits in patients with HFpEF, HFrEF, patients with diabetes, obesity, chronic kidney disease, or those using semaglutide or dulaglutide, while liraglutide use showed a neutral effect. Drug-related side effects were monitored as safety outcomes, which showed no significant differences between groups.

**Conclusions:**

In ASCVD and HF patients, adding GLP-1 RA to SGLT2i reduces 1-year mortality and hospitalization, warranting further investigation in diverse settings.

## Introduction

Atherosclerotic cardiovascular disease (ASCVD) continues to be a major contributor to morbidity and mortality, with many patients suffering from concurrent conditions such as heart failure (HF), which complicates clinical management.^1^^,^^2^ ASCVD is widely recognized as a major contributing factor to HF, responsible for ∼70% of cases.^3^ Moreover, there is a strong association between ASCVD and diabetes mellitus. Diabetes mellitus is linked to ∼two-fold increased risk of ASCVD, independent of other conventional risk factors.^4^ Patients with diabetes who develop ASCVD face a significantly higher risk of mortality.^5^ Fortunately, novel anti-diabetic agents, including sodium-glucose cotransporter 2 inhibitors (SGLT2i) and glucagon-like peptide-1 receptor agonists (GLP-1 RA), have demonstrated significant cardiovascular benefits.^6–8^ Both SGLT2i and GLP-1 RA have been shown to reduce major adverse cardiovascular events (MACE) in patients with ASCVD, while SGLT2i has also been proven to improve outcomes in patients with HF.^9^^,^^10^ However, the optimized treatment strategies for patients with ASCVD and HF remain inadequately defined.

In previous randomized controlled trials (RCTs), SGLT2i has shown a trend towards improving MACE in patients with both ASCVD and HF, suggesting its potential benefit in this population.^11–15^ Similarly, two major GLP-1 RA trials have indicated potential clinical benefits in this cohort; however, these findings were derived from subgroup analyses with a limited number of patients.^16^^,^^17^ Despite these promising trends, it remains unclear whether the combined use of GLP-1 RA and SGLT2i can further enhance clinical outcomes in patients with both ASCVD and HF compared with SGLT2i treatment alone.

Given the significant overlap between ASCVD and HF in clinical practice, addressing this knowledge gap is essential for optimizing treatment strategies. To address this, our study aims to leverage the TriNetX database to compare the outcomes of patients treated with GLP-1 RA in addition to SGLT2i vs. those treated with SGLT2i alone, with a specific focus on hard endpoints such as mortality and HF hospitalizations.

## Methods

### Data Source

This retrospective observational study utilized data from the TriNetX database, which aggregates de-identified, patient-level information from electronic health records. The TriNetX database sources its data from health care organizations (HCOs), primarily academic medical centres, which include primary hospitals, affiliated satellite hospitals, and outpatient clinics. The available data include patient demographics, diagnoses (coded using the International Classification of Diseases, 10th revision, clinical modification [ICD-10-CM]), procedures (classified by ICD-10 procedure coding system or current procedural terminology), medications (based on Veterans Affairs Drug Classification System and RxNorm codes), laboratory tests (using logical observation identifiers names and codes [LOINC]), and health care utilization records. For this study, we used data from the Global Collaborative Network within TriNetX, which encompasses information from over 124 million patients across 131 HCOs and 15 countries.^18^

The results were validated using independent, industry-standard methods, and summarized for investigators. Further details about the database are available online^19^ and have been previously described in the literature.^18^

Ethical approval for this study utilizing the TriNetX database was obtained from the institutional review board of Chi Mei Hospital (11310-E03). As the study relied solely on aggregated statistical summaries of de-identified data, the requirement for informed consent was waived. This study was conducted in accordance with the principles outlined in the Declaration of Helsinki^20^ and followed the Strengthening the Reporting of Observational Studies in Epidemiology reporting guidelines.^21^

### Cohort

This retrospective study included patients aged 18 years or older with ASCVD and HF who were newly prescribed GLP-1 RA and SGLT2i or SGLT2i alone. Data were collected from the TriNetX database, covering an 8-year period between 1 August 2016 and 30 September 2024. The start date aligns with the publication of the LEADER trial.^22^ The patients were divided into two groups, GLP-1 RA with SGLT2i and SGLT2i alone groups. In the SGLT2i alone group, patients previously prescribed GLP-1 RA were excluded. Patients who died or were hospitalized within one week of initiating GLP-1 RA were excluded. We performed 1:1 propensity score matching (PSM) using 37 variables, including demographics, diagnoses, and medications. Patients in both groups were followed for up to 1 year. Details regarding the codes used to identify demographics, diagnoses, and medications are provided in Supplementary material online, *Table S1*.

### Covariables

Covariate selection was guided by clinical relevance, focusing on major comorbidities and risk factors that could influence mortality, hospitalization, or cardiovascular outcomes based on current knowledge. The following variables were considered to adjust for imbalances in baseline characteristics between the GLP-1 RA with SGLT2i and SGLT2i alone group: age, sex, race, ethnicity (as recorded in the electronic health record), respiratory disease, circulatory disease, endocrine or metabolic disease, nutritional disease, diabetes mellitus, medications such as angiotensin-converting enzyme inhibitor, angiotensin II receptor blocker, beta blocker, spironolactone, eplerenone, nitrates, vericiguat, calcium channel blocker, vasodilators, ivabradine, ranolazine, digitalis glycosides, HMA CoA reductase inhibitors, ezetimibe, evolocumab, alirocumab, aspirin, clopidogrel, ticagrelor, prasugrel, cangrelor, furosemide, clinical variables, including left ventricular ejection fraction (LVEF), NT-proBNP, HbA1c, and troponin I. Further details on the categorization and the codes used to define the covariates are available in *Table 1*.

**Table 1:** Baseline Characteristics of the GLP-1RA with SGLT2i and SGLT2i alone groups before and after propensity score matching

	Before matching, No. (%)			After matching, No. (%)		
Characteristics	GLP-1RA with SGLT2i (*n* = 5548)	SGLT2i alone (*n* = 90 503)	Standardized difference	GLP-1RA with SGLT2i (*n* = 5272)	SGLT2i alone (*n* = 5272)	Standardized difference
Age, mean(SD), y	63.7 ± 12	68.4 ± 12.8	0.3790	63.7 ± 12	63.8 ± 13	0.0036
Sex						
Female	1392 (33.0%)	21 135 (30.1%)	0.0623	1390 (32.9%)	1364 (32.3%)	0.0132
Male	2685 (63.6%)	45 309 (64.5%)	0.0185	2684 (63.7%)	2715 (64.6%)	0.0153
Race and ethnicity						
White	2552 (60.5%)	41 248 (58.7%)	0.0356	2552 (60.5%)	2564 (60.8%)	0.0058
Black or African American	745 (17.6%)	10 950 (15.6%)	0.0555	743 (17.6%)	746 (17.7%)	0.0019
Hispanic or Latino	276 (6.5%)	4354 (6.2%)	0.0140	276 (6.5%)	269 (6.3%)	0.0068
Asian	256 (6.0%)	3610 (5.1%)	0.0403	256 (6.0%)	246 (5.8%)	0.0100
Not Hispanic or Latino	3222 (76.4%)	48 618 (69.2%)	0.1610	3219 (76.4%)	3 252 (77.1%)	0.0186
Diagnoses						
Diseases of the respiratory system	2486 (58.9%)	42 653 (60.7%)	0.0372	2484 (58.9%)	2513 (59.6%)	0.0140
Endocrine, nutritional and metabolic diseases	4131 (97.9%)	64 175 (91.4%)	0.2950	4128 (97.9%)	4126 (97.9%)	0.0034
Diabetes mellitus	3821 (90.6%)	37 196 (53.1%)	0.9206	3818 (90.6%)	3822 (90.7%)	0.0033
Metabolic disorders	3751 (88.9%)	58 212 (82.9%)	0.1737	3748 (88.9%)	3762 (89.2%)	0.0107
Symptoms and signs involving the circulatory and respiratory systems	2782 (65.9%)	46 201 (65.8%)	0.0030	2779 (65.9%)	2772 (65.7%)	0.0035
Medications						
Beta blocking agents	3 568 (84.6%)	58 229 (82.9%)	0.0447	3565 (84.6%)	3598 (85.4%)	0.0219
HMG CoA reductase inhibitors	3558 (84.3%)	54 397 (77.5%)	0.1756	3555 (84.3%)	3567 (84.6%)	0.0079
Aspirin	2799 (66.3%)	45 538 (64.8%)	0.0314	2798 (66.4%)	2812 (66.7%)	0.0070
Furosemide	2599 (61.6%)	46 335 (66.0%)	0.0914	2598 (61.6%)	2570 (61.0%)	0.0136
Angiotensin II receptor blockers, plain	2445 (57.9%)	40 198 (57.2%)	0.0143	2443 (57.9%)	2418 (57.3%)	0.0120
Organic nitrate nitrates	1934 (45.8%)	29 529 (42.0%)	0.0764	1933 (45.8%)	1903 (45.1%)	0.0143
Calcium channel blockers	1660 (39.3%)	27 505 (39.1%)	0.0036	1658 (39.3%)	1675 (39.7%)	0.0083
Spironolactone	1657 (39.3%)	28 004 (39.9%)	0.0124	1655 (39.2%)	1 608(38.1%)	0.0229
Clopidogrel	1341 (31.8%)	19 253 (27.4%)	0.0958	1341 (31.8%)	1267 (30.0%)	0.0380
Angiotensin II receptor blockers (ARBs), combinations	1300 (30.8%)	20 964 (29.8%)	0.0208	1299 (30.8%)	1299 (30.8%)	<0.0001
ACE inhibitors, plain	1268 (30.0%)	20 037 (28.5%)	0.0334	1268 (30.0%)	1248(29.6%)	0.0104
Ezetimibe	503 (11.9%)	5792 (8.2%)	0.1223	500 (11.8%)	486 (11.5%)	0.0103
Ticagrelor	355 (8.4%)	5198 (7.4%)	0.0375	354 (8.4%)	369 (8.7%)	0.0127
Digitalis glycosides	303 (7.1%)	5285 (7.5%)	0.0132	303(7.1%)	313(7.4%)	0.0091
Ranolazine	211 (5.0%)	2220 (3.1%)	0.0931	211 (5.0%)	213(5.0%)	0.0022
Eplerenone	140 (3.3%)	2047 (2.9%)	0.0232	140 (3.3%)	125(2.9%)	0.0204
Prasugrel	131 (3.1%)	1424 (2.0%)	0.0682	130 (3.0%)	133(3.1%)	0.0041
Evolocumab	88 (2.0%)	638 (0.9%)	0.0971	85 (2.0%)	82(1.9%)	0.0051
Ivabradine	53 (1.2%)	761 (1.0%)	0.0160	53 (1.2%)	46(1.0%)	0.0154
Alirocumab	36 (0.8%)	243 (0.3%)	0.0658	34 (0.8%)	36(0.8%)	0.0052
Cangrelor	20 (0.4%)	400 (0.5%)	0.0133	20 (0.4%)	20(0.4%)	<0.0001
Other vasodilators used in cardiac diseases	18 (0.4%)	283 (0.4%)	0.0037	17	18	0.0037
Vericiguat	14 (0.3%)	113 (0.1%)	0.0345	13(0.3%)	12(0.2%)	0.0044
Left ventricular ejection fraction (%)	43.4 ± 15	39.2 ± 15	0.2662	43.4 ± 15	39.3 ± 15	0.0228
NT pro-BNP (pg/mL)	3286 ± 6.1	5545 ± 7.9	0.3188	3290 ± 6.1	5 022 ± 7.5	0.0061
Hba1c (%)	8.2 ± 2.1	6.8 ± 1.7	0.7478	8.2 ± 2.1	7.5 ± 1.9	0.0098
Troponin I (ug/L)	2.0 ± 9.6	3.3 ± 18.3	0.0945	2.0 ± 9.6	3.1 ± 15.1	0.0030

### Primary and secondary outcomes

The primary outcome was a composite of mortality or hospitalization occurring within 1 year from the initiation of treatment. Secondary outcomes included individual measures of mortality, hospitalization, and heart failure exacerbation (HFE) during the same 1-year period. HFE was defined using ICD-10-CM codes, intravenous diuretics, or a diagnosis of pulmonary edema in emergency or inpatient settings.^23^

### Negative control outcomes, subgroup, and sensitivity analysis

To establish a baseline for comparison, acute cholecystitis, fracture, and gastric ulcer were used as negative control outcomes. We conducted pre-specified subgroup analyses based on HF type (HFrEF or HFpEF), the presence of diabetes mellitus, obesity, and chronic kidney disease (CKD) to identify specific subgroups that might influence the observed outcomes. Additionally, we used different types of GLP-1 RA (liraglutide, semaglutide, and dulaglutide) to determine whether the observed benefits were consistent across different medications.

To evaluate the robustness of our findings, we conducted a sensitivity analysis by extending the follow-up period to 2 and 3 years and using different sets of variables in the PSM analysis, including separate analyses excluding drug variables, diagnosis variables, and demographic variables such as age, sex, and ethnicity. To evaluate the generalizability of the study findings across different racial and ethnic groups, sensitivity analyses were conducted stratified by race (Black, White, and Asian) and ethnicity (Hispanic, non-Hispanic, or Latino). We also conducted sensitivity analyses stratified by GLP-1 RA dose, and adherence. Adherence was defined as the continued use of medication from 6 months to 1 year after treatment initiation, assessed through prescription refill records. To further evaluate the clinical benefits of combination therapy, we conducted a sensitivity analysis comparing combination therapy with GLP-1 RA monotherapy. To address potential bias introduced by including patients with contraindications to GLP-1 RA, we conducted a sensitivity analysis excluding these patients. Contraindications of GLP-1 RA were identified as medullary thyroid carcinoma, malignant neoplasm of thyroid gland, multiple endocrine neoplasia syndrome, and acute pancreatitis.

Intermediate markers were evaluated to provide additional insights into the mechanisms underlying the observed outcomes. These markers included LVEF, NT-proBNP, body mass index (BMI), and fasting glucose.

### Safety outcomes

Drug-related side effects, such as urinary tract infection, diabetic ketoacidosis, acute pancreatitis, gastroparesis, and intestinal obstruction, were recorded and summarized in a supplementary table. The incidence of these adverse events was compared between the GLP-1 RA combined with SGLT2i group and the SGLT2i alone group to provide detailed insights into treatment safety profiles.

### Statistical analysis

Baseline characteristics of the two groups were presented as means with standard deviations (SDs) for continuous variables or as counts with percentages for categorical variables. Categorical variables were compared using the *χ*² test, while continuous variables were compared using an independent 2-sample *t*-test. One-to-one PSM was conducted using the greedy nearest neighbour algorithm with a caliper of 0.1 pooled SDs to balance baseline characteristics between the two groups. Adequate matching was achieved when the standardized difference between groups was <0.1. Survival probabilities after PSM were calculated using the Kaplan–Meier method. Hazard ratio (HR) with 95% confidence intervals (CIs) and *P*-values were calculated using Cox proportional hazards regression models for all outcomes. The potential impact of unmeasured confounding was evaluated using the E-value method, which estimates the minimum strength of association that an unmeasured confounder would need to explain the observed differences between the two groups. An E-value of *x* indicates that the observed association could only be attributed to an unmeasured confounder with a risk ratio of at least x-fold for both the treatment and outcome, beyond the effects of the measured confounders (*Table 2*).^24^

**Table 2:** Comparison of GLP-1RA with SGLT2i vs. SGLT2i alone for primary and secondary outcomes

	Number of patients with outcomes				
Outcome	GLP-1RA with SGLT2i (*n* = 5272)	SGLT2i alone (*n* = 5272)	HR(%CI)	*P* value	*E* value
Primary outcome					
Mortality or hospitalization	2162	2436	0.78 (0.74–0.83)	<0.0001	1.66
Secondary outcome					
Mortality	321	408	0.72 (0.62–0.84)	<0.0001	2.12
hospitalization	2044	2321	0.78 (0.73–0.83)	<0.0001	1.66
Heart failure exacerbation	1294	1535	0.77 (0.72–0.83)	<0.0001	1.69

All statistical tests were two-sided, with a significance level of *P* < 0.05. Statistical analyses were performed using the TriNetX platform's analytic tool.

## Results

**Figure 1:**
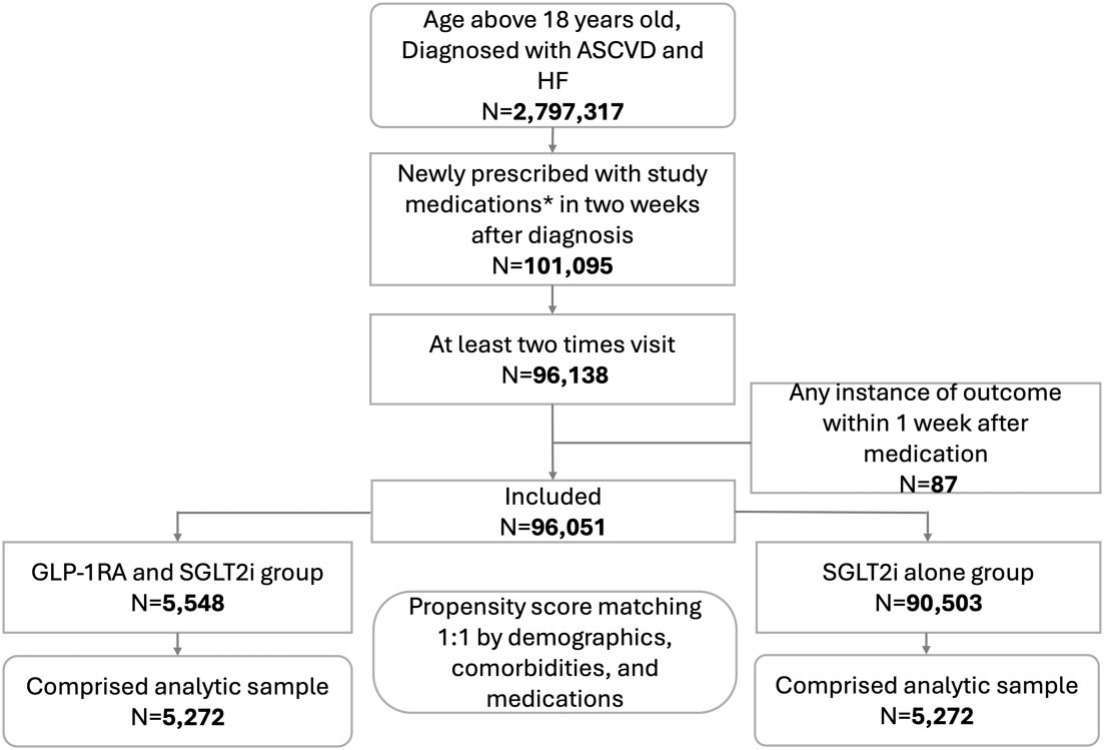
Flow diagram of cohort construction. Abbreviations: ASCVD, atherosclerotic cardiovascular disease; HF, heart failure; GLP-1 RA, glucagon-like peptide-1 receptor agonist; SGLT2i, sodium–glucose transport protein 2 inhibitors. *Study medications: GLP-1 RA with SGLT2i or SGLT2i alone.

A total of 2 797 317 patients over the age of 18 were diagnosed with ASCVD and HF between 1 August 2016 and 30 September 2024. Among them, 101 095 patients initiated GLP-1 RA combined with SGLT2i or SGLT2i alone within two weeks of diagnosis. After excluding those with fewer than two visits or outcomes occurring within one week of starting medication, we included 96 051 patients in the final analysis. Of these,  5548 patients received both GLP-1 RA and SGLT2i, while 90 503 received SGLT2i alone. Based on demographics, comorbidities, and concurrent medications, PSM resulted in 5272 patients in each group (*Figure 1*).

Before PSM, patients in the GLP-1 RA and SGLT2i group were younger than those in the SGLT2i alone group (63.7 ± 12 vs. 68.4 ± 12 years, *P* < 0.0001). The proportion of males was similar between the groups (63.6% vs. 64.5%, *P* = 0.2428). GLP-1 RA and SGLT2i group had a higher percentage of Black or African American (17.6% vs. 15.6%, *P* = 0.0003) and Asian patients (6.0% vs. 5.1%, *P* = 0.0084) compared to SGLT2i alone group. Patients in the GLP-1 RA and SGLT2i group had higher prevalence of endocrine disease (97.9% vs. 91.4%, *P* < 0.0001), diabetes mellitus (90.6% vs. 53.0%, *P* < 0.0001), metabolic disease (88.9% vs. 82.9%, *P* < 0.0001) but a lower percentage in disease of the respiratory system (58.9% vs. 60.7%, *P* = 0.0187) compared to those in the SGLT2i alone group. Before PSM, patients in the GLP-1 RA and SGLT2i group were more likely to use beta-blockers (84.6% vs. 82.9%, *P* = 0.0057), HMA CoA reductase inhibitors (84.3% vs. 77.5%, *P* < 0.0001), Aspirin (66.3% vs. 64.8%, *P* = 0.0486), nitrate (45.8% vs. 42.0%, *P* < 0.0001), Clopidogrel (31.8% vs. 27.4%, *P* < 0.0001), angiotensin-converting enzyme inhibitors (30.0% vs. 28.5%, *P* = 0.0340), ezetimibe (11.9% vs. 8.2%, *P* < 0.0001), Ticagrelor (8.4% vs. 7.4%, *P* = 0.0151), Ranolazine (5.0% vs. 3.1%, *P* < 0.0001), Prasugrel (3.1% vs. 2.0%, *P* < 0.0001), Evolocumab (2.0% vs. 0.9%, *P* < 0.0001), Alirocumab (0.8% vs. 0.3%, *P* < 0.0001), and Vericiguat (0.3% vs. 0.1%, *P* < 0.0001) but were less likely to use Furosemide (61.6% vs 66.0%, *P* < 0.0001) compared to those in the SGLT2i alone group. Before PSM, the GLP-1 RA and SGLT2i group exhibited higher LVEF (43.4 ± 15 vs. 39.2 ± 15%, *P* < 0.0001) and HbA1c (8.2 ± 2.1 vs. 6.8 ± 1.7%, *P* < 0.0001) levels but lower NT-proBNP (3286 ± 6.1 vs. 5545 ± 7.9 pg/mL, *P* < 0.0001) levels compared to the SGLT2i alone group. Additionally, troponin I levels were similar between the two groups prior to PSM (2.0 ± 9.6 vs. 3.3 ± 18.3 ug/L, *P* = 0.7608). After PSM, baseline characteristics were well-balanced between the groups, with no significant differences (p > 0.05) (*Table 1*) (Supplementary material online, *Figure S1*). Before and after matching of the propensity score density curves are shown in the Supplementary (Supplementary material online, *Figure S1*).

### Primary outcome

**Figure 2:**
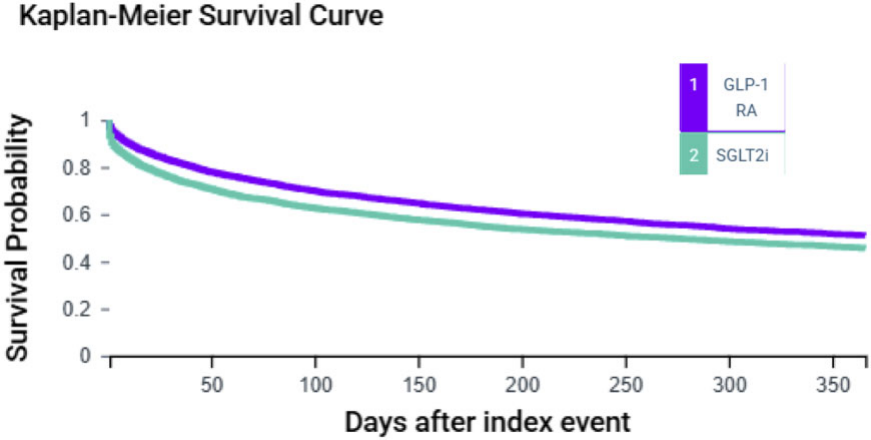
This Kaplan–Meier survival curve compares patients with ASCVD and HF treated with GLP-1 RA combined with SGLT2i vs. those treated with SGLT2i alone. The GLP-1 RA and SGLT2i group demonstrate a significantly lower event rate over the 1-year follow-up compared to the SGLT2i alone group. ASCVD, atherosclerotic cardiovascular disease; HF, heart failure; GLP-1 RA, glucagon-like peptide-1 receptor agonist; SGLT2i, sodium-glucose transport protein 2 inhibitors.

Within the first year, 2162 patients in the GLP-1 RA and SGLT2i group and 2462 in the SGLT2i alone group experienced mortality or hospitalization. The incidence of mortality or hospitalization was significantly lower in the GLP-1 RA and SGLT2i group compared with the SGLT2i alone group (HR 0.78; 95% CI 0.74–0.83; *P* < 0.0001) (*Table 2* and *Figure 2*).

### Secondary outcome

The risk of mortality (HR 0.72; 95% CI 0.62–0.84; *P* < 0.0001), hospitalization (HR 0.78; 95% CI 0.73–0.83; *P* < 0.0001) and HFE (HR 0.77; 95% CI 0.72–0.83, *P* < 0.0001) were significantly lower in patients who received both GLP-1 RA and SGLT2i compared to those who received SGLT2i alone (*Table 2*). E-value analysis indicated that unmeasured confounders were unlikely to significantly affect our findings, with E-values for the point estimates being 1.66 for mortality or hospitalization, 2.12 for mortality, 1.66 for hospitalization, and 1.69 for HFE, respectively (*Table 2*).

### Negative control outcomes, subgroup, and sensitivity analysis

Negative control analysis revealed no significant difference between the GLP-1 RA and SGLT2i group and the SGLT2i alone group for acute cholecystitis, fracture, or gastric ulcer (Supplementary material online, *Table S2*). The results of this study were consistent across subgroups, including HFrEF, HFpEF, the presence or absence of diabetes mellitus, obesity or CKD, and the use of semaglutide or dulaglutide (Supplementary material online, *Table S2*). Additionally, patients with obesity exhibited better outcomes than those without obesity (interaction *P* = 0.0365). However, liraglutide use did not demonstrate significant benefits in subgroup analysis (HR 0.89; 95% CI 0.78–1.02).

In sensitivity analysis, the outcome benefits persisted at 2- and 3-year follow-ups and were consistent when using different sets of variables in the PSM analysis (Supplementary material online, *Table S2*). In another sensitivity analysis stratified by race and ethnicity, the combination therapy consistently demonstrated a reduced risk compared to SGLT2i alone in most groups. [Black (HR 0.67, 95% CI 0.58–0.78); White (HR 0.84, 95% CI 0.78–0.90); Asian (HR 0.73, 95% CI 0.57–0.93); and Not Hispanic or Latino (HR 0.77, 95% CI 0.72–0.82)]. However, in the Hispanic group, the association did not reach statistical significance (HR 0.92, 95% CI 0.74–1.15) (Supplementary material online, *Table S2*). Patients receiving semaglutide 1.34 mg/mL (HR 0.78, 95% CI 0.68–0.89) and dulaglutide 1.5 mg/mL (HR 0.81, 95% CI 0.71–0.92) demonstrated statistically significant lower composite outcomes compared to SGLT2i alone group. However, no significant differences were observed in groups using other different doses of GLP-1 RA. In sensitivity analyses restricted to patients with consistent medication adherence, the findings remained consistent with the overall results (HR 0.82, 95% CI 0.67–0.99). The combination therapy demonstrated a significant association with improved outcomes compared to GLP-1 RA monotherapy (HR 0.89, 95% CI 0.84–0.95) (Supplementary material online, *Figure S2*). After excluding patients with contraindications to GLP-1 RA in the sensitivity analysis, the findings were consistent with the primary results (HR 0.80, 95% CI 0.76–0.85).

In the analysis of intermediate markers, the combination therapy group had significantly higher LVEF (44.5% vs. 41.8%, *P* = 0.0019) and BMI (33.5 vs. 30.7 kg/m^2^, *P* < 0.0001) compared to the SGLT2i alone group. Additionally, NT-proBNP levels (3594 vs. 5067 pg/mL, *P* = 0.0002) were significantly lower in the combination therapy group. Fasting glucose levels were similar between groups (147 vs. 151 mg/dL, *P* = 0.6275) (Supplementary material online, *Table S3*).

### Safety outcomes

Drug-related side effects, including urinary tract infection, diabetic ketoacidosis, acute pancreatitis, gastroparesis, and intestinal obstruction, were evaluated in both groups. No significant differences were observed between the GLP-1 RA and SGLT2i group and the SGLT2i alone group (Supplementary material online, *Table S4*).

## Discussion

Our research found that adding GLP-1 RA to SGLT2i therapy in patients with ASCVD and HF was associated with lower 1-year mortality and hospitalization rates compared to SGLT2i alone. Secondary outcomes, including mortality, hospitalization, and HFE showed consistent results. Concordant results were observed across subgroup analyses, including patients with HFpEF, HFrEF, diabetes, obesity, CKD, and those receiving semaglutide, liraglutide, or dulaglutide. Subgroup analysis revealed that combination therapy was particularly effective in obese patients compared to non-obese patients. However, no significant reduction in mortality or hospitalization was observed in the liraglutide subgroup. This finding is consistent with prior studies indicating that semaglutide offers superior weight and glycemic control compared to liraglutide.^25^^,^^26^ Mechanistically, semaglutide's longer half-life, combined with its less frequent dosing schedule, enhances patient adherence and provides more sustained therapeutic effects compared to liraglutide.^27^^,^^28^ Moreover, semaglutide has been shown to exert stronger central nervous system actions, directly targeting GLP-1 receptors in the brain, which may contribute to its superior efficacy.^29^

Sensitivity analysis demonstrated sustained benefits at 2- and 3-year follow-ups, and across different sets of PSM covariates. Sensitivity analyses stratified by race and ethnicity showed consistent results across most groups. However, the Hispanic group did not reach statistical significance. The outcome remained consistent when comparing combination therapy to the GLP-1 RA monotherapy group. The sensitivity analysis excluding patients with contraindications to GLP-1 RA further supports the robustness of our findings. Significant differences in intermediate markers, particularly LVEF and NT-proBNP, might suggest potential synergistic effects of GLP-1 RA and SGLT2i on cardiac remodelling and myocardial stress reduction.^30^^,^^31^ These findings warrant further investigation to elucidate the underlying mechanisms. The safety profile of GLP-1 RA combined with SGLT2i therapy was comparable to that of SGLT2i monotherapy, with no significant differences observed in the incidence of adverse events, including urinary tract infection, diabetic ketoacidosis, acute pancreatitis, gastroparesis, and intestinal obstruction.

Previous research primarily focuses on the benefit of medications targeting individual comorbidity.^32^ GLP-1 RA has been shown to be effective in ASCVD patients, while SGLT2i proved to be effective in patients with HF or ASCVD.^8^^,^^10^ However, in the clinical setting, these comorbidities often co-exist and interact in ways that can amplify disease progression and complicate management. For instance, the presence of HF in patients with ASCVD can exacerbate cardiovascular stress and increase the risk of hospitalization, while conditions such as diabetes and obesity can further contribute to adverse outcomes by promoting inflammation and endothelial dysfunction. The interconnected pathophysiology underscores the need for comprehensive treatment strategies addressing multiple comorbidities to optimize outcomes.^33^^,^^34^

In patients with ASCVD and HF, subgroup analyses of previous RCTs suggest that both GLP-1 RA and SGLT2i may offer potential benefits compared to placebo.^11–17^ However, these trials included relatively small subgroups of patients with both ASCVD and HF, and these outcomes were not the primary focus of the studies. Furthermore, the benefits of combining these two drugs remain unclear. Our research adds to the growing evidence supporting the benefits of these therapies in patients with both ASCVD and HF.

Patients with ASCVD and HF may have underlying pathophysiology associated with ischaemic cardiomyopathy. Ischaemia can impair heart function through mechanisms such as endothelial dysfunction, inflammation, calcium homeostasis disruption, and mitochondrial dysfunction.^2^^,^^35^^,^^36^ GLP-1 RA may improve outcomes by regulating calcium levels through reduced ryanodine receptor 2 phosphorylation and activation of calmodulin-dependent protein kinase II.^37–39^ Additionally, GLP-1 receptor signalling may inhibit oxidative stress and mitochondrial dysfunction in cardiac cells via the GLP-1R/cAMP/Epac/PI3K/Akt pathway.^40^ These mechanisms may underlie the observed endpoint benefits of GLP-1 RA in this patient population, highlighting their potential to improve clinical outcomes in this population.

In the current era, coronary artery bypass surgery is the only treatment proven to reduce mortality in patients with ischaemic cardiomyopathy, while other medical therapies primarily address HF management.^41^ Our research suggests a potential new potential therapeutic option to improve prognosis in patients with both ASCVD and HF.

### Limitation

This study has several limitations. First, as TriNetX data are registry-based, there may be issues with misidentification and underrepresentation, particularly for milder cases or individuals not engaged with the healthcare system, which could impact the results. Additionally, the lack of detailed demographic data limits our ability to account for geographic and socioeconomic disparities, lifestyle factors, and healthcare access, potentially impacting the generalizability of our findings. To address this limitation, we performed sensitivity analyses stratified by race and ethnicity to strengthen our findings. The use of diagnostic codes to identify variables and outcomes may also lead to misclassification, potentially introducing bias. To address this potential information bias, we conducted a negative control analysis to compare unrelated events with GLP-1 RA and found no significant differences, suggesting minimal registration bias. Second, the primary outcomes were assessed over 1 year. Although sensitivity analyses with 2-year and 3-year follow-up data showed consistent results, the long-term effects of combination therapy on prognosis remain unclear. Further studies with extended follow-up periods are needed to fully understand the sustained benefits and risks of GLP-1 RA and SGLT2i therapy. Third, unmeasured variables may have influenced the outcomes, introducing potential confounding. Given the observational design of this study and baseline differences between groups, residual confounding cannot be entirely ruled out. To address this, we calculated E-values. Higher E-values exceeding the HRs suggest that minor unaccounted confounders would be insufficient to nullify the observed association, thereby strengthening the robustness of our conclusions. However, the inherent limitations of an observational study preclude the establishment of definitive causality, which requires further validation through prospective studies or RCTs. Finally, due to database limitations, we were unable to specify the cause of mortality and hospitalization, which may have introduced additional bias. To address this limitation, we included HFE as a secondary outcome to provide additional insights and partially mitigate this issue.

## Conclusion

In summary, the addition of GLP-1 RA to SGLT2i therapy significantly reduces 1-year mortality and hospitalization rates in patients with ASCVD and HF compared to SGLT2i alone. These findings suggest that combined GLP-1 RA and SGLT2i therapy may offer a valuable treatment approach to improve outcomes in this high-risk population. Further research is warranted to validate these benefits in broader clinical settings.

## Supplementary Material

pvaf014_Supplemental_File

## Data Availability

Data described in the manuscript, code book, and analytic code will be made publicly and freely available without restriction at https://trinetx.com.
