# Supplementary material for: Prognostic benefit of glucagon-like peptide-1 receptor agonists addition to sodium-glucose cotransporter 2 inhibitors in patients with atherosclerotic cardiovascular disease and heart failure: a cohort study
Source: Eur Heart J Cardiovasc Pharmacother. 2025 Feb 17;11(4):324–33. doi: 10.1093/ehjcvp/pvaf014 (PMC12231126; doi:10.1093/ehjcvp/pvaf014)
Supplement: pvaf014_Supplemental_File [file pvaf014_supplemental_file.docx]

Supplementary Table 1: Demographic, Diagnostic, and Laboratory Codes Used in the Definition of Covariates and outcomes

| Category | Code | Description |
| --- | --- | --- |
| Demographics | AI | Age at Index |
| Demographics | M | Male |
| Demographics | F | Female |
| Demographics | 2106-3 | White |
| Demographics | 2186-5 | Not Hispanic or Latino |
| Demographics | 2054-5 | Black or African American |
| Demographics | 2028-9 | Asian |
| Demographics | 2135-2 | Hispanic or Latino |
| Diagnoses | E00-E89 | Endocrine, nutritional and metabolic diseases |
| Diagnoses | E08-E13 | Diabetes mellitus |
| Diagnoses | E70-E88 | Metabolic disorders |
| Diagnoses | R00-R09 | Symptoms and signs involving the circulatory and respiratory systems |
| Diagnoses | J00-J99 | Diseases of the respiratory system |
| Diagnoses | I00-I99 | Diseases of the circulatory system |
| Medications | C07 | Beta blockers |
| Medications | C10AA | HMG CoA reductase |
| Medications | 1191 | Aspirin |
| Medications | 4603 | Furosemide |
| Medications | C09C | Angiotensin II antagonists, plain |
| Medications | C01DA | Organic nitrate nitrates |
| Medications | 9997 | Spironolactone |
| Medications | C08 | Calcium channel blocker |
| Medications | 32968 | Clopidogrel |
| Medications | C09D | Angiotensin II antagonists, combinations |
| Medications | C09A | ACE inhibitors, plain |
| Medications | 341248 | Ezetimibe |
| Medications | 1116632 | Ticagrelor |
| Medications | C01AA | Digitalis glycosides |
| Medications | 35829 | Ranolazine |
| Medications | 298869 | Eplerenone |
| Medications | 613391 | Prasugrel |
| Medications | 1665684 | Evolocumab |
| Medications | 1649480 | Ivabradine |
| Medications | 1659152 | Alirocumab |

| Supplementary Table 1 (continued) | | |
| --- | --- | --- |
| Category | Code | Description |
| Medications | 1656052 | Cangrelor |
| Medications | C01DX | Other vasodilators used in cardiac diseases |
| Medications | 2475830 | Vericiguat |
| Medications | F | Female |
| Outcomes | | |
| All-cause mortality | UMLS:ICD10CM:R99 | Ill-defined and unknown cause of mortality |
|  | Deceased | Deceased |
| All-cause hospitalization | Inpatient encounter | Inpatient encounter |
|  | Inpatient non-acute | Inpatient non-acute |
|  | Observation encounter | Observation encounter |
| Heart failure exacerbation | CV702 | LOOP DIURETICS [Injectable Product] |
|  | RxNorm:6916 | metolazone |
|  | UMLS:ICD10CM:J81 | Pulmonary edema |
|  | CV701 | THIAZIDES/RELATED DIURETICS [Injectable Product] |
| GLP-1 RA contraindication | 8345/3 | Medullary thyroid carcinoma |
|  | C73 | Malignant neoplasm of thyroid gland |
|  | E31.2 | Multiple endocrine neoplasia syndromes |
|  | K85 | Acute pancreatitis |

Supplementary Table 2: negative control, subgroup, and sensitivity analysis

|  | Hazard ratio | 95% CI | |  |
| --- | --- | --- | --- | --- |
| Negative control outcomes | | | | |
| Acute cholecystitis | 0.83 | 0.48-1.44 | |  |
| Fracture | 0.87 | 0.57-1.32 | |  |
| Gastric ulcer | 1.22 | 0.82-1.80 | |  |
| Subgroup analysis | | | | |
| ASCVD and HFrEF | 0.79 | 0.73-0.86 | |  |
| ASCVD and HFpEF | 0.87 | 0.79-0.96 | |  |
| ASCVD and HF with no DM | 0.81 | 0.70-0.94 | Interaction p=0.4578 |  |
| ASCVD and HF with DM | 0.76 | 0.71-0.82 |  |  |
| ASCVD and HF with obesity | 0.73 | 0.66-0.82 | Interaction p=0.0365 |  |
| ASCVD and HF without obesity | 0.84 | 0.78-0.91 |  |  |
| ASCVD and HF with CKD | 0.73 | 0.66-0.81 | Interaction p=0.2060 |  |
| ASCVD and HF without CKD | 0.79 | 0.74-0.85 |  |  |
| GLP-1 RA with Liraglutide | 0.89 | 0.78-1.02 | |  |
| GLP-1 RA with Semaglutide | 0.76 | 0.70-0.83 | |  |
| GLP-1 RA with Dulaglutide | 0.76 | 0.69-0.83 | |  |
| Sensitivity analysis | | | | |
| Black | 0.67 | 0.58-0.78 | |  |
| White | 0.84 | 0.78-0.90 | |  |
| Hispanic | 0.92 | 0.74-1.15 | |  |
| Asian | 0.73 | 0.57-0.93 | |  |
| Not Hispanic or Latino | 0.77 | 0.72-0.82 | |  |
| Follow up two years | 0.83 | 0.78-0.88 | |  |
| Follow up three years | 0.82 | 0.78-0.87 | |  |
| Result with no drug PSM | 0.78 | 0.73-0.82 | |  |
| Result with no diagnosis PSM | 0.85 | 0.80-0.90 | |  |
| Result with no age, sex and ethnicity PSM | 0.80 | 0.75-0.84 | |  |
| Semaglutide 0.68 mg/ml | 1.00 | 0.84-1.19 | |  |
| Semaglutide 1.34 mg/ml | 0.78 | 0.68-0.89 | |  |
| Liraglutide 3.6 mg/ml | 1.57 | 0.52-4.69 | |  |
| Liraglutide 6 mg/ml | 1.11 | 0.95-1.30 | |  |
| Dulaglutide 1.5 mg/ml | 0.81 | 0.71-0.92 | |  |
| Dulaglutide 3.0 mg/ml | 0.93 | 0.75-1.16 | |  |
| Drug adherence | 0.82 | 0.67-0.99 | |  |
| Combination therapy vs GLP-1 RA alone | 0.89 | 0.84-0.95 | |  |
| Exclude patients with GLP-1 RA contraindication | 0.80 | 0.76-0.85 | |  |

Supplementary Table 3: Laboratory data distribution

|  | GLP-1RA with SGLT2i | SGLT2i alone | P value |
| --- | --- | --- | --- |
| LVEF(%) | 44.5 | 41.8 | 0.0019 |
| NT-pro BNP(pg/mL) | 3,594 | 5,067 | 0.0002 |
| BMI(kg/m^2^) | 33.5 | 30.7 | <0.0001 |
| Fasting glucose(mg/dL) | 147 | 151 | 0.6275 |

| Side effects | GLP-1RA with SGLT2 | SGLT2i alone | P value |
| --- | --- | --- | --- |
| Urinary tract infection | 179 | 205 | 0.08 |
| Diabetic ketoacidosis | 18 | <10 | 0.17 |
| Acute pancreatitis | 24 | 17 | 0.23 |
| Gastroparesis | 36 | 25 | 0.19 |
| Intestine obstruction | 16 | 29 | 0.99 |

Supplementary Table 4: safety outcomes

Supplementary Figure legend


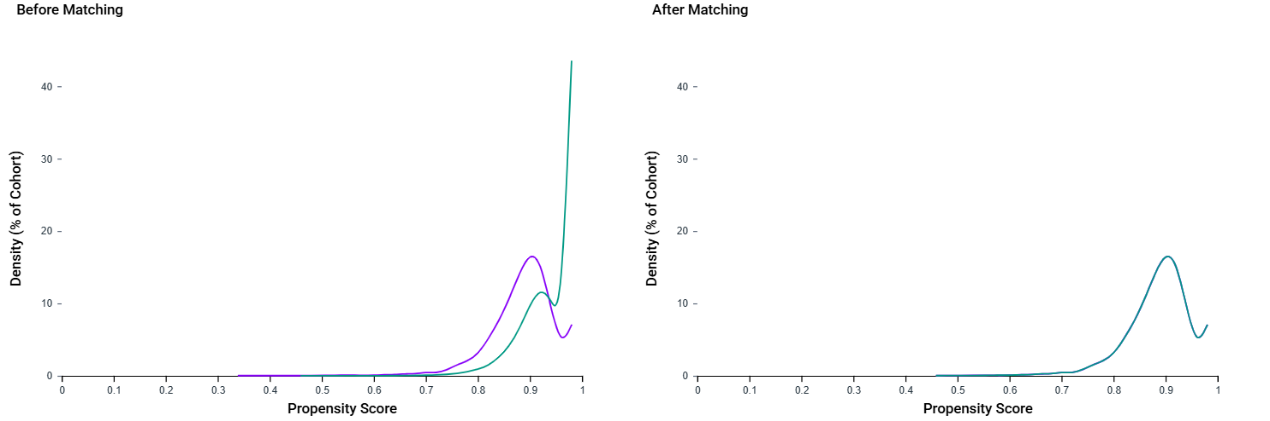
Supplementary Figure 1: the pre- and post-propensity score matching density curves
